# Hydrochalcogenation Reactions with Noncanonical Amino Acids as a Route to Increase Bioconjugate Valency

**DOI:** 10.1021/acsomega.5c07015

**Published:** 2025-10-02

**Authors:** Emily L. Boyt, Tyler L. Skeen, Cedrick R. Dimaranan, Evan M. London, Aaron S. Wang, Sophia K. Rothman, Milania G. Dehring, Alexander C. Willard, Emily M. Peairs, Elizabeth A. King, Douglas D. Young

**Affiliations:** Department of Chemistry, 8604 William & Mary, Williamsburg, Virginia 23185, United States

## Abstract

Bioconjugation represents an efficient and rapid mechanism to enhance the already extensive utility of proteins; however, it requires the development of additional chemistries to prevent undesired reactivity with nascent biological functionalities. Moreover, the ability to conjugate multiple partners to increase the conjugate valency further expands potential applications. Herein, we describe the development and optimization of a thio-yne bioconjugation using rongalite coupled with noncanonical amino acids (ncAAs) to afford multivalent conjugates. The site-specificity of the ncAA partner provides a functional handle to perform a biological Glaser-Hay reaction that installs the functionality for a secondary hydrochalcogenation. These trivalent conjugates serve as a proof-of-concept for the development of potent therapeutic and diagnostic agents that have vast applicability.

## Graphical abstract

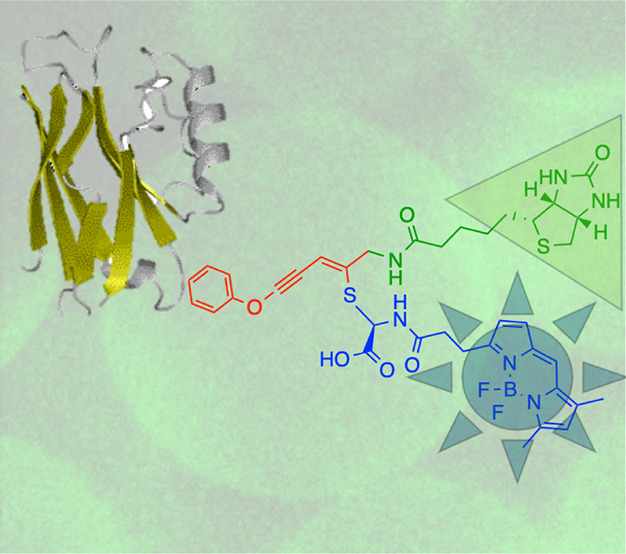

## Introduction

Protein bioconjugates, or proteins that have been covalently functionalized with a probe, surface, synthetic polymer, or additional biomolecule, have wide chemical and biological applications.^1–3^ Such conjugates have advanced the fields of materials chemistry, therapeutics, diagnostics, molecular imaging, and numerous others.^4–11^ For instance, antibody-drug conjugates (ADCs), or cytotoxic agents attached to a cancer-targeting monoclonal antibody, have emerged as a more potent and directed form of cancer therapeutics.^12–20^ Additionally, the conjugation of spectroscopic probes, stabilizing polymers, or purification tags to proteins can facilitate the study of protein functionality and biological processes in *in vivo* and *in vitro* settings.^6,21–27^ In many applications of protein bioconjugation, it is advantageous, if not essential, to generate site-specific, homogeneous conjugates. Initial methodologies in the field relied on either nonspecific conjugation at nucleophilic residues or modification of protein termini to introduce novel functionality.^28^ Each of these approaches has its own distinct drawbacks.^15,29–31^ For example, precise control over the number and locations of drug molecules attached to an antibody is crucial to ensure the safety and efficacy of ADCs.^4,16^ One method to address selectivity issues involves the incorporation of noncanonical amino acids (ncAAs) into proteins.^32–34^ Specifically, ncAAs bearing bioorthogonal functionalities can be introduced into proteins at predetermined residues using techniques such as genetic code expansion.^35–39^ These ncAAs can then serve as unique reaction handles in bioconjugation. Given the breadth of genetic code expansion technologies, numerous bioorthogonal ncAAs have been synthesized, incorporated, and utilized to generate functional bioconjugates with a precise level of control.^32,40–48^

The majority of existing bioconjugation techniques allow a single reaction partner to be coupled to a protein, forming a divalent bioconjugate. The utility of protein bioconjugates, however, could be further enhanced through the synthesis of multivalent protein bioconjugates, in which two or more different reaction partners are attached to a protein.^29,49–51^ The therapeutic value of these multivalent conjugates is vast, including attaching multiple differently acting therapeutics to a targeting protein or the addition of a serum-stabilizing agent to enhance not only delivery but also improve pharmacokinetic properties.^27^ Multivalent proteins would also have distinct advantages in diagnostic applications. Metabolite-binding proteins could be immobilized with an additional probe that facilitates quantification of protein surface attachment to enhance measurement accuracy.^26,52–54^ Moreover, protein–protein/metabolite associations could be investigated via conjugating both a pull-down tag and a fluorescent probe, which affords both visualization and purification of protein–protein interactions to further characterize these interactions and their genomic roles.^46,55,56^

Unfortunately, methods for the conjugation of multiple molecules to a protein are extremely limited, mostly due to the challenge of identifying multiple unique conjugation handles within a protein. A common approach to forming multivalent proteins involves attaching multiple copies of a protein, as well as numerous types of small molecules, to a polymeric scaffold such as a dendrimer.^4,24,51^ While numerous multivalent conjugates have been synthesized in this manner, including many conjugates for therapeutic and/or diagnostic applications, polymer-based multivalent conjugates often suffer from heterogeneity in both the number and spatial distribution of attached ligands. This polymeric approach possesses low conjugation efficiency and high variability, resulting in decreased efficacy.^24,57^

The employment of ncAAs has the potential to circumvent some of the issues in the preparation of multivalent bioconjugates. One direct mechanism is via the incorporation of two unique ncAAs into the protein via further genetic code expansion with multiple repurposed codons or the utilization of a 4-base codon.^58–61^ While these approaches have distinct utility, they require significant genetic manipulation, increase protein structural perturbation, lower conjugate yields, and take longer to prepare. These approaches have found distinct use in the preparation of ADCs and other therapeutics.^62–64^

A more direct route to prepare multivalent conjugates that requires less genetic infrastructure and affords more rapid access to conjugates involves the utilization of a single ncAA. Only a handful of approaches to synthesizing multivalent protein bioconjugates using a single ncAA have previously been explored.^55,65,66^ Recently, a bifunctional tetrazine/azide ncAA was incorporated into a protein for dual functionalization with an inverse-electron-demand Diels–Alder reaction and a strain-promoted 1,3-dipolar cycloaddition reaction, respectively.^66^ Though promising, these reactions could potentially be limited by cross-reactivity between their substrates, hampering the approach’s homogeneity and specificity. Previous work in our laboratory elucidated a cascade reaction approach utilizing a *p*-bromopropargyloxyphenylalanine ncAA that could first be coupled via a copper­(I)-catalyzed azide–alkyne cycloaddition (CuAAC) to yield a halo-triazole.^65^ This aromatic halide is then primed to undergo a bioorthogonal Sonogashira coupling with a terminal alkyne.^67,68^ This approach successfully afforded a functional multivalent conjugate; however, the use of potentially cytotoxic transition metal catalysts in both steps of the synthesis is less than ideal. Consequently, this work seeks to expand the available chemical toolbox to prepare multivalent conjugates using a single ncAA. Namely, we leverage our previously developed bioorthogonal Glaser-Hay coupling of terminal alkynes to generate a reactive diyne that is primed to undergo a second reaction to introduce new functionality into the conjugate.^69–72^ Specifically, we intended to capitalize on well-established thio-yne chemistries to perform hydrochalcogenations under physiological conditions and yield multivalent conjugates.

## Materials and Methods

### General

Solvents and reagents, including BODIPY FL l-cystine, were obtained from Sigma-Aldrich, Fisher Scientific, or VWR and used without further purification. Plasmids were obtained from the laboratory of Dr. Peter Schultz at The Scripps Research Institute. Reactions were conducted under ambient atmosphere with solvents directly from the manufacturer. All proteins were purified according to the manufacturer’s protocols using a Qiagen Ni-NTA Quik Spin Kit. SDS-PAGE was performed using a BioRad Mini-PROTEAN Tetra system and visualized on a BioRad gel imaging system.

### Expression of *p*-Propargyloxyphenylalanine (*p*PrF)-Containing Green Fluorescent Protein (GFP) and Ubiquitin (Ub)

Using an Eppendorf electroporator set to 1800 V, *Escherichia coli* BL21 (DE3) cells were cotransformed with a pET-GFP-TAG-151 or pET-Ub-TAG-48 plasmid (0.5 μL) and the polyspecific pEVOL-*p*CNF plasmid (0.5 μL) and allowed to recover for 1 h in LB media at 37 °C. Following recovery, the cells were plated on an LB agar plate supplemented with ampicillin (50 μg/mL) and chloramphenicol (34 μg/mL) and grown at 37 °C for 16 h. Next, a single colony from the plate was used to inoculate 5 mL of LB media, also supplemented with ampicillin and chloramphenicol. The culture was grown to confluence at 37 °C for 16 h. The dense culture was used to begin an expression culture in LB media (25 mL) supplemented with ampicillin and chloramphenicol at an OD_600_ of 0.1. The expression culture was incubated at 37 °C to an OD_600_ of 0.7–0.9, then induced with IPTG (1 M, 25 μL), arabinose (20%, 25 μL), and the *p*PrF ncAA (100 mM, 250 μL). The cells were then incubated at 37 °C for 16 h and pelleted by centrifugation (10 min, 5000 rpm), after which the supernatant was discarded and the pellet was stored at −80 °C for 20 min. The cell pellet was then resuspended and lysed with BugBuster (VWR) for 30 min at 37 °C and centrifuged to remove cellular debris, and the lysate containing the protein was isolated. Protein was then purified using a Qiagen Ni-NTA Quik Spin Kit according to the manufacturer’s protocol. Protein yield and purity were assessed via SDS-PAGE and spectrophotometrically via a Nanodrop spectrophotometer. Finally, the proteins were buffer-exchanged into phosphate-buffered saline (PBS; 10 mM Na_2_HPO_4_, 2 mM KH_2_PO_4_, 2.7 mM KCl, 137 mM NaCl, pH 6) using concentration columns (5 kDa MWCO for ubiquitin and 10 kDa MWCO for GFP, Corning Spin-X).

### Glaser-Hay Bioconjugation of GFP

CuI (7.2 mg, 0.04 mmol) and TMEDA (76 μL, 0.46 mmol) were combined in DI water (302 μL). This mixture was then sonicated and heated at 60 °C for 15 min. After heating, the mixture was vortexed and cooled on ice. Next, *p*PrF-harboring protein (25 μL, ∼1 mg/mL, PBS pH 6) and catalase (7.5 μL, 9 mg/mL, PBS pH 6) were added to a PCR tube. The CuI/TMEDA mixture was vortexed, and 5 μL of the CuI/TMEDA mixture were added to the PCR tube. The PCR tube was then heated at 37 °C for 15 min. The alkyne partner (12.5 μL, 1 mM; i.e., biotin alkyne, fluorophore alkyne, etc.) was added to the PCR tube. The tube was sealed, and the reaction was incubated at room temperature for 4 h. Finally, the reaction was concentrated and buffer-exchanged using a spin column (10 kDa MWCO, Corning Spin-X; PBS: 10 mM Na_2_HPO_4_, 2 mM KH_2_PO_4_, 2.7 mM KCl, 137 mM NaCl, pH 6). The reaction mixture was washed with additional PBS (8 × 200 μL) and concentrated to a final volume of 25 μL. The protein conjugate was analyzed by the biotin immobilization assay and SDS-PAGE to assess the bioconjugation efficiency.

### General Hydrochalcogenation Bioconjugation Procedure

Sodium hydroxymethanesulfinate (rongalite; 38.5 mg, 0.25 mmol) was weighed into an Eppendorf tube and dissolved in DI H_2_O (1 mL). K_2_CO_3_ (21.8 mg, 0.16 mmol) was added to a separate Eppendorf tube and dissolved in DI H_2_O (1 mL). The rongalite solution (100 μL) and the K_2_CO_3_ solution (100 μL) were then combined and diluted with DI H_2_O (800 μL). This solution (20 μL) was combined with either GFP-biotin Glaser-Hay conjugate or GFP-*p*PrF (25 μL, ∼1 mg/mL, PBS pH 6) and either BODIPY FL l-cystine (5 μL, 1 mM) or FITC-labeled selenocystamine (5 μL, ∼3.25 mM) in a PCR tube. The reaction was incubated for 24 h at room temperature. Finally, the reaction was added to a concentrator column (5 kDa or 10 kDa MWCO, Corning Spin-X) that was hydrated with PBS (10 mM Na_2_HPO_4_, 2 mM KH_2_PO_4_, 2.7 mM KCl, 137 mM NaCl, pH 6). The reaction mixture was washed with additional PBS (8 × 200 μL) and then concentrated to a final volume of 25 μL. The protein conjugate was analyzed by SDS-PAGE to assess bioconjugation.

### Biotin Immobilization Assay

Immobilized streptavidin resin (G-Biosciences; 30 μL) was transferred to a 0.5 mL PCR tube. The resin was pelleted using a tabletop minifuge with 30 s pulses (Eppendorf), and the supernatant was removed. The resin was then equilibrated twice by resuspension in PBS (50 μL; 10 mM Na_2_HPO_4_, 2 mM KH_2_PO_4_, 2.7 mM KCl, 137 mM NaCl, pH 6), followed by pelleting and removal of the supernatant. Next, either the GFP-biotin conjugate (3 μL), ubiquitin-biotin-fluorophore conjugate (10 μL), or GFP-Alexa Fluor 488 conjugate (10 μL; negative control) was added directly to the resin pellet. The tube was sealed and incubated for 30 min at room temperature. The resin was then washed with PBS (10 × 50 μL) by using the previously described pelleting procedure to remove any unbound protein. The beads were then imaged with a BIO-RAD ZOE fluorescent cell imager to assess their fluorescence and biotin–streptavidin interaction.

## Results and Discussion

Given our prior success in the development and optimization of a bioorthogonal Glaser-Hay coupling, we hypothesized that the rigid, linear, and electron-rich 1,3-diyne functionality could serve as an excellent secondary site to introduce additional functionality. With the rich literature precedence of thio-yne additions, we hypothesized that this could be a fruitful route to multivalency.^73–76^ While initially optimizing the biological Glaser-Hay conjugation, we examined the diyne moiety with thiol reagents to ensure bioorthogonality, given the presence of numerous biological thiols (e.g., cysteine, glutathione). In the absence of any catalyst or additive, we did not observe reactivity with the 1,3-diyne.^72^ This observation confirmed the physiological stability of the divalent conjugate but did not exclude the potential for additional reactivity under the appropriate catalytic conditions.

To initiate our investigations into the potential of thio-yne multivalent additions, we first prepared divalent conjugates utilizing ncAAs. Model proteins GFP and ubiquitin were expressed harboring the *p*PrF ncAA at tyrosine residue 151 or lysine residue 48, respectively, using standard genetic code expansion technologies (Figure 1).^77^ Conveniently, this is a common ncAA that is easily synthesized and already has an evolved synthetase/tRNA pair for incorporation. Following protein purification and SDS-PAGE analysis to confirm successful amber codon suppression with *p*PrF, we subjected the protein to optimized Glaser-Hay coupling conditions using a CuI/TMEDA cocatalyst and either an AlexaFluor-488-alkyne or a biotin-alkyne partner to establish the divalent protein conjugate (Figure 1, see Supporting Information). Successful conjugation was confirmed by MS, SDS-PAGE, and streptavidin bead assays. Prior to incubation with the beads, the GFP was denatured to eliminate natural fluorescence, allowing fluorescence to be observed only when coupled to a fluorophore. While conjugation of either protein with a fluorophore alkyne facilitated fluorescent confirmation of the reaction (see Supporting Information), reaction with the biotin alkyne and ubiquitin afforded no rapid functional assay to assess coupling, as there was no fluorescence and ubiquitin is not enzymatic. However, the success of all other conjugations was used to infer productive reaction of the two species, and future experiments were able to validate this assumption.

**Figure 1:**
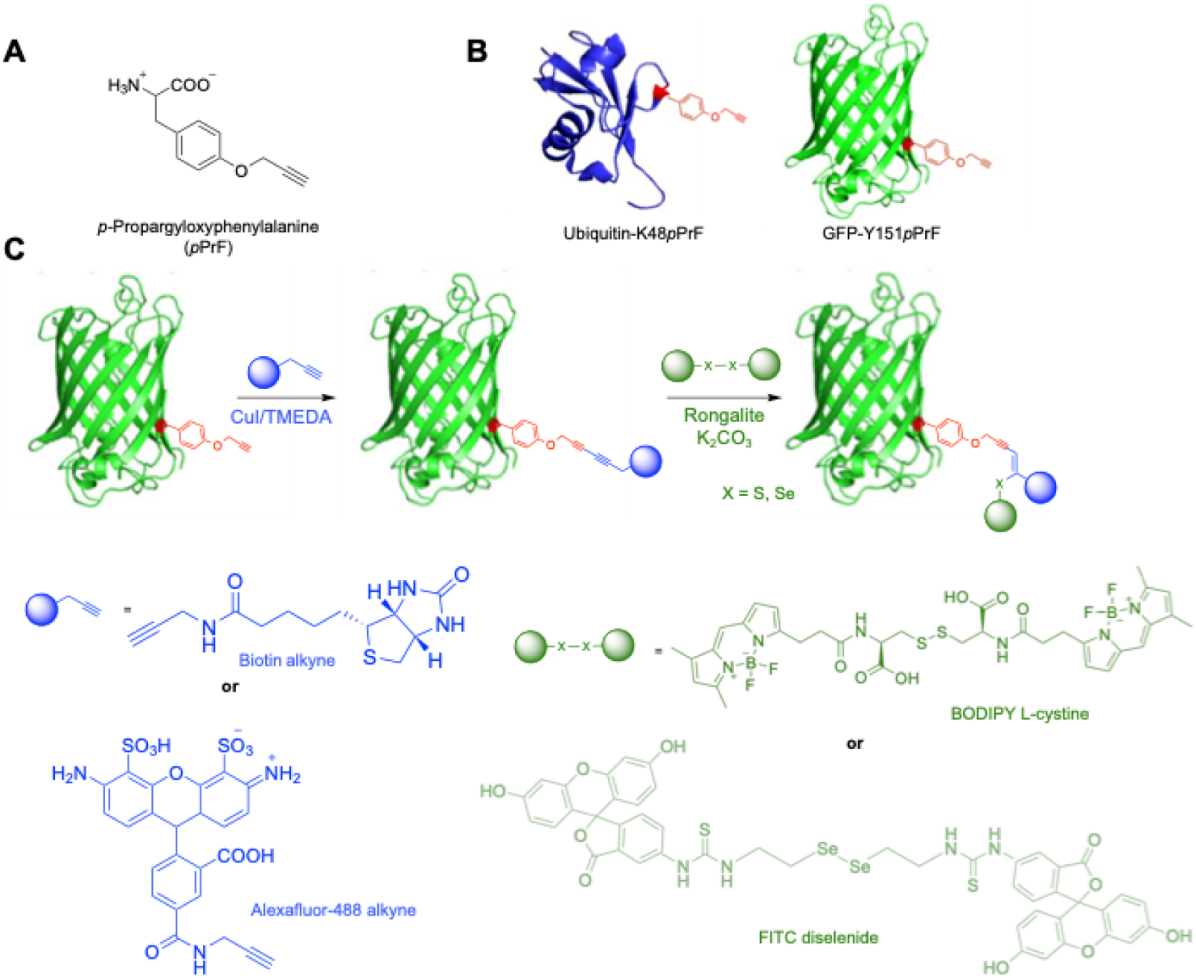
Proposed biological hydrochalcogenation conjugation scheme and components. A) *p*-propargyloxyphenylalanine (*p*PrF) a noncanonical amino acid (ncAA), is incorporated into the protein for use in bioconjugation. B) Crystal structure representation of the *p*PrF ncAA incorporated into the ubiquitin protein, replacing a lysine at residue 48 (blue protein), and the GFP protein, replacing a tyrosine at residue 151 (green protein). C) Proposed route to multivalency. An initial Glaser-Hay coupling of two terminal alkynes was performed using either an AlexaFluor-488 alkyne partner or a biotin alkyne in the presence of Cu/TMEDA with the ubiquitin-K48*p*PrF or GFP-Y151*p*PrF protein. The conjugated protein can be further elaborated via reaction with either a BODIPY l-cystine fluorophore or FITC diselenide using a rongalite-based alkyne hydrothiolation/selenation to make a trivalent conjugate.

With the divalent 1,3-diyne conjugate prepared, thio-yne addition could be investigated as a mechanism to introduce a third functional group to the conjugate. Our initial investigations involved ubiquitin-linked proteins and the introduction of a BODIPY FL l-cystine fluorophore. Based on literature reports, reactions were examined using either 2-hydroxy-4’-(2-hydroxyethyl)-2-methylpropiophenone (I2959) or 2,2-dimethoxy-2-phenylacetophenone (DPAP) as photoinitiators.^73,78^ Divalent conjugates and cystine fluorophore were combined in PBS (pH 7.2) at various concentrations and irradiated with either 302 or 365 nm light for 0–30 min, and then allowed to react postirradiation for 0–24 h. Reactions were then screened for productivity via denaturing SDS-PAGE analysis for the fluorescent trivalent conjugates. While some fluorescence was observed (see Supporting Information), productive reactions required high loadings of photoinitiator, long UV irradiation times of 30 min, and long reaction times. Moreover, even under the most intensive conditions, the reactions were determined to reach only 50–60% completion based on fluorescence. These results prompted a reevaluation of coupling conditions and further probing of the literature.

A more recent report identified the preparation of (*Z*)-sulfanylenynes and (*Z*)-selanylenynes by the reaction of 1,3-diynes with diaryl disulfides and diaryl diselenides in the presence of the reducing agent sodium hydroxymethanesulfinate (rongalite) and the base K_2_CO_3_.^79^ These reactions are believed to proceed via a radical-based mechanism and have been performed at low temperatures and in aqueous media, making them suitable for bioconjugations (Scheme 1). Furthermore, a wide variety of 1,3-diynes, including dialkyl-, diaryl-, and alkyl/aryl-substituted, could be utilized. This suggests that the internal 1,3-diyne formed through the Glaser-Hay coupling of a terminal alkyne-containing protein and a terminal alkyne probe could be a suitable substrate for these reactions. It is important to note that in these reactions and subsequent bioconjugation reactions there is not exclusive regioselectivity; however, only one regioisomer is depicted. Consequently, the focus of the research transitioned to optimizing these conditions for preparing multivalent conjugates.

**Scheme 1:**
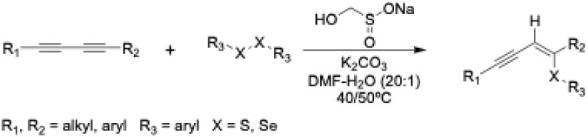


Initially, the previously described GFP-biotin conjugate (∼1 mg/mL, PBS pH 6) was combined with BODIPY FL l-cystine (4.5 mM), rongalite (0.16 mM), and K_2_CO_3_ (0.18 mM) and allowed to react at room temperature for ∼24 h (Scheme 2). After this time, unreacted fluorophore was removed via buffer exchange with a molecular weight cutoff spin column. SDS-PAGE followed by fluorescence imaging revealed the presence of a fluorescent band at ∼27 kDa, suggesting that the hydrothiolation reaction occurred but in low yield. It is important to note that the fluorescence is due to a productive reaction, rather than GFP fluorescence, as control reactions in the absence of appropriate conditions exhibited no fluorescence due to denaturing conditions. However, the presence of a trivalent conjugate, with mild reaction conditions and without UV irradiation, indicated the value of the rongalite addition and promoted further optimization of conditions. The ratio of the rongalite/K_2_CO_3_ radical initiator was reevaluated, and ultimately a 3:2 molar ratio was found to be ideal, matching prior reports in the literature.^79–81^ It is important to note that the rongalite/K_2_CO_3_ initiator needs to be prepared freshly for each reaction and that rongalite is prone to decomposition over time, resulting in decreased coupling efficiencies.

**Scheme 2:**
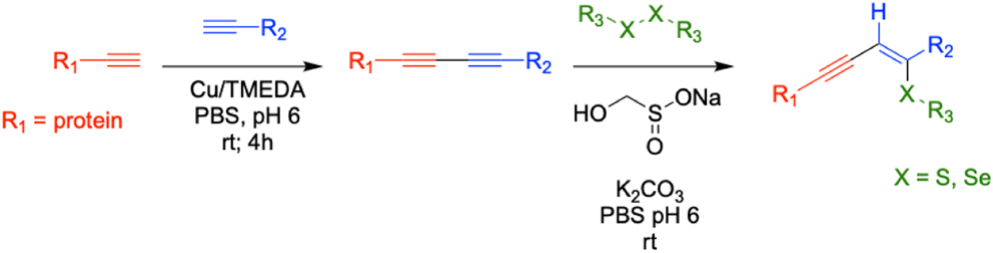


Several other reaction conditions were next optimized, including pH, temperature, and the addition of a reducing agent (dithiothreitol, DTT).^82^ The GFP-biotin conjugate (∼1 mg/mL, PBS pH 6, 7, or 8) was combined with BODIPY FL l-cystine (0.2 mM). A rongalite/K_2_CO_3_ (10 mM/6.6 mM) solution was prepared and added to initiate the reaction. The reaction was then allowed to proceed in either the presence or absence of DTT (1 mM) at varying temperatures. After 24 h, samples were subjected to SDS-PAGE with fluorescence imaging to measure reaction success (Figure 2A). This analysis revealed that pH 6 and room temperature conditions were optimal for the hydrothiolation of the 1,3-diyne conjugate. Interestingly, elevated temperatures resulted in lower conjugation yields. Furthermore, the presence of DTT was found to exert no positive influence on reaction success and can be withheld from the reaction (Supporting Information). Control experiments were also performed with both WT GFP and WT ubiquitin at room temperature in the presence of both the fluorophore and rongalite/K_2_CO_3_. Gratifyingly, no protein labeling was observed, confirming that the alkynyl functionality must be present for the reaction and that nonspecific fluorophore labeling does not occur (see Supporting Information).

**Figure 2:**
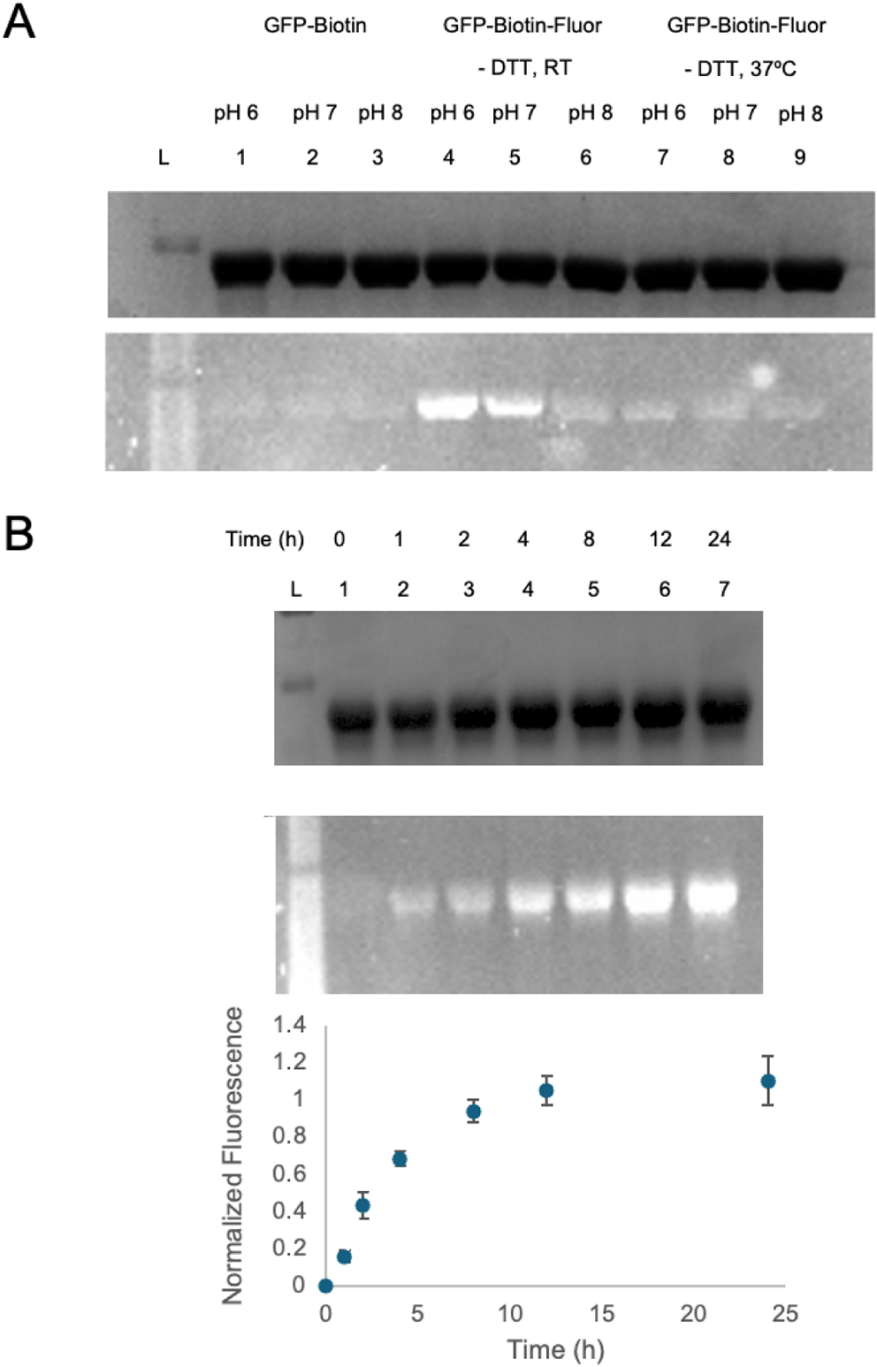
Optimization of the rongalite-assisted thio-yne multivalent bioconjugation. A) Screening of pH and temperature in the hydrothiolation bioconjugation reaction. An SDS-PAGE fluorescent image (bottom) and subsequent Coomassie blue-stained image (top) of the rongalite multivalent bioconjugate optimization screen. The GFP-biotin divalent conjugate was reacted with BODIPY FL l-cystine (0.2 mM), rongalite (10 mM), and K_2_CO_3_ (6.6 mM). The optimization screen indicates that the most promising conditions for the reaction occur at room temperature with a pH of 6 in the absence of the reducing agent DTT. B) SDS-PAGE fluorescence (bottom) and subsequent stain (top) of a timecourse screening of the hydrothiolation bioconjugation using optimal conditions. The reaction appears to run to completion at 12 h with no detectable protein degradation. Densitometry measurements of the fluorophore fluorescence relative to the densitometry measurement of the total protein band at each given time interval (bottom graph) confirm qualitative analysis of reaction completion at 12 h. Trials were performed in triplicate to provide the standard deviation.

Finally, in order to fully characterize the utility of the reaction, we used the optimal conditions to perform a time-course experiment. While the reaction is not as rapid as some of the recently developed cycloaddition chemistries, the thio-yne addition appears to be complete after approximately 12 h at room temperature, with no detectable protein degradation (Figure 2B). Control experiments measuring WT-GFP fluorescence prior to the reaction and post-reaction demonstrate no notable change, indicating minimal degradation and loss of function. However, it is important to note that GFP is a relatively hardy protein, and translation of the reaction to less stable proteins will require a reassessment of protein function/degradation. Calculating protein concentration through densitometry/BCA assays and using fluorophore absorbance measurements to calculate fluorophore concentration via Beer’s Law, afforded the determination of conjugation yield at 12 h to be 94% and 97% after 24 h.^72^ This is especially advantageous, as this approach also does not require transition metal catalysts known to degrade proteins. The trivalent conjugate was also confirmed by mass spectrometry to be the expected mass, given the addition of a single chalcogen substituent, signifying that only one addition occurs under these conditions (see Supporting Information).

Given the successful optimization of the thiol-based reaction, we next sought to elucidate the feasibility of performing similar conjugation reactions with selenides based on the organic reactions reported in the literature. In addition to the ability to introduce novel functionality to the bioconjugate, the presence of a selenium atom in the conjugate may have further advantages given its antioxidant properties.^83–85^ However, unlike thiol-based reagents that are commonly employed in biochemical reactions, fewer commercially available selenide reagents, especially fluorophores, are easily obtainable. Toward our efforts to develop the hydroselenation bioconjugation, we opted to prepare a diselenide probe. Using literature procedures with oxidized glutathione, initial attempts to prepare a fluorescent probe based on the reaction of FITC with l-selenocystine afforded a probe.^86^ However, the unique chemical properties, solubility, and p*K*_a_ of the selenocystine resulted in the inability to drive reactions to completion and isolate the pure product. Additionally, reagent degradation became preventative in advancing the amino acid base toward conjugation reactions. Given that many of these issues were attributed to the l-selenocystine starting material, we instead transitioned to selenocystamine dihydrochloride to simplify the synthesis. Utilizing similar synthetic conditions in a carbonate buffer (pH 10), the selenocystamine was reacted with FITC at room temperature for 3 days, resulting in the consumption of all FITC and starting material to create a mixture of mono- and di-labeled diselenide (see Supporting Information) as observed by TLC. Following column chromatography and analysis by mass spectrometry, it was feasible to isolate the doubly labeled product that could be employed in conjugation reactions (see Supporting Information). A GFP-biotin Glaser-Hay conjugate (∼1 mg/mL, PBS pH 6) was obtained and combined with the diselenide fluorophore (0.33 mM), rongalite (10 mM), and K_2_CO_3_ (6.6 mM) in a manner analogous to the optimized hydrothiolation bioconjugation. Following buffer exchange, the reaction mixture was analyzed via SDS-PAGE to reveal the successful preparation of a selenide trivalent conjugate (Scheme 2 and Figure 3). Control experiments with WT GFP and WT ubiquitin not harboring an alkyne functionality resulted in no labeling, demonstrating that nonspecific labeling of the selenide compound does not occur under these conditions (see Supporting Information).

**Figure 3:**
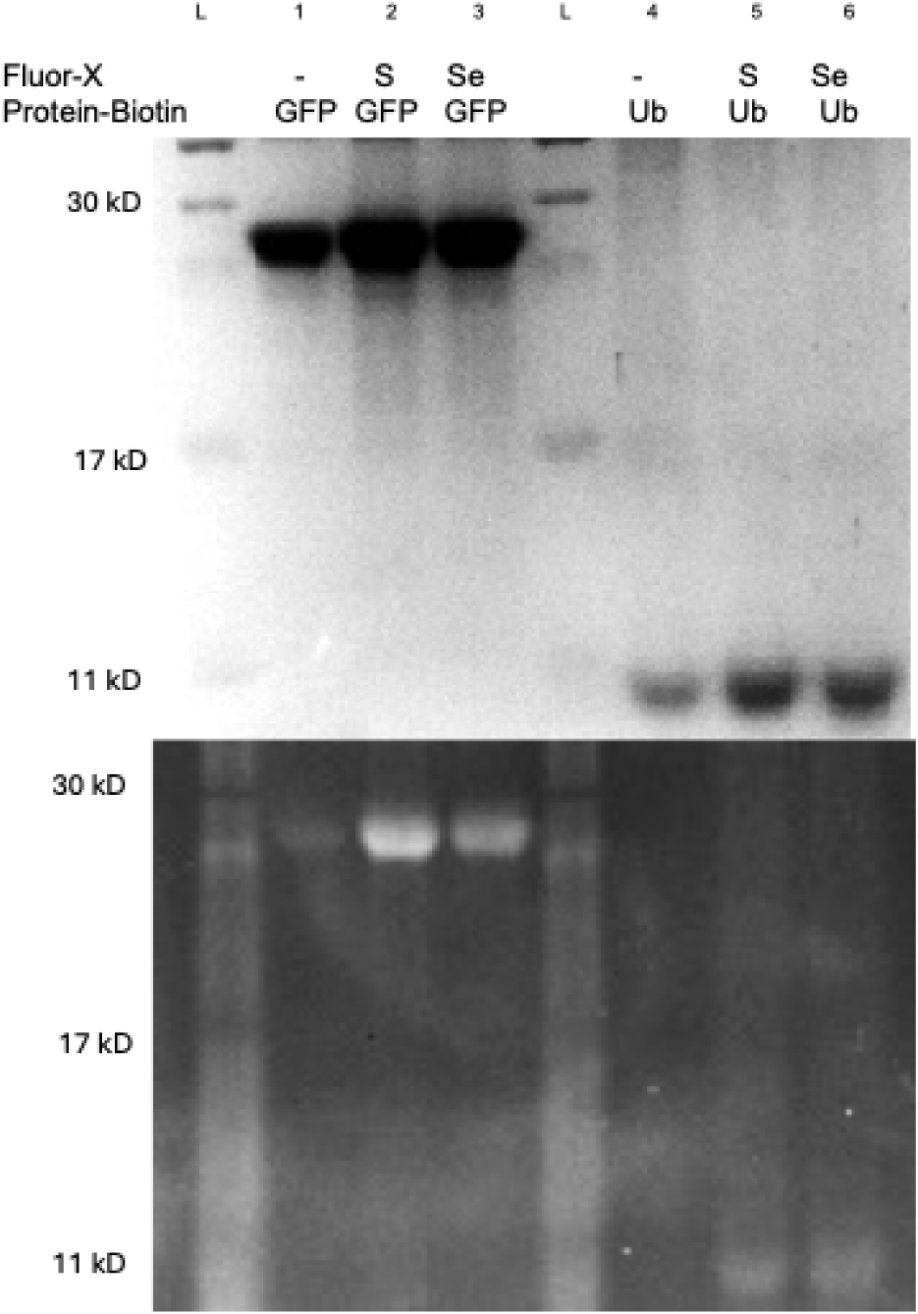
SDS-PAGE depicting multivalent protein conjugates of either GFP-151-biotin or Ub-48-biotin reacting with disulfide (BODIPY FL l-cystine) or diselenide (FITC diselenide) fluorophores. Coomassie blue staining (top) confirms the presence of GFP in lanes 1–3 and ubiquitin proteins in lanes 4–6. The fluorescence (bottom) demonstrates fluorophore conjugation in lanes 2–3 and 5–6, in which the divalent protein-biotin conjugate is treated with BODIPY FL l-cystine or FITC diselenide fluorophores. Reactions treated with rongalite in the absence of a fluorophore (lanes 1 and 4) contained no fluorescent protein. (S: BODIPY FL l-cystine; Se: FITC diselenide).

Finally, we aimed to demonstrate a proof-of-concept to illustrate the utility of multivalency. Using our prepared ubiquitin-biotin-fluorophore conjugates, we performed a streptavidin pull-down assay to not only confirm the successful cascade reaction but also act as a mechanism to rapidly purify conjugates. Trivalent conjugates were incubated with streptavidin-bound resin to leverage the strong binding interaction between biotin and streptavidin. After ten washes of the resin with PBS, the resin was examined by fluorescence microscopy to confirm the presence of fluorescent conjugates (Figure 4). Gratifyingly, fluorescence was observed only in the beads incubated with the trivalent conjugate and was not a result of nonspecific fluorophore interactions when incubated only with the fluorophore or with a divalent conjugate that did not harbor biotin. This result confirms that both the Glaser-Hay reaction and the thio-yne reaction were successful, as both reactions are necessary for binding and subsequent fluorescence. While this is only a proof-of-concept for the application of multivalent conjugates, the experiment represents a useful application in both pull-down experiments and the immobilization of proteins. As previously demonstrated by our laboratory, this protein immobilization is useful in various diagnostic techniques and in the ability to increase the stability of proteins and employ protein catalysis in nonaqueous solvents.^53^

**Figure 4:**
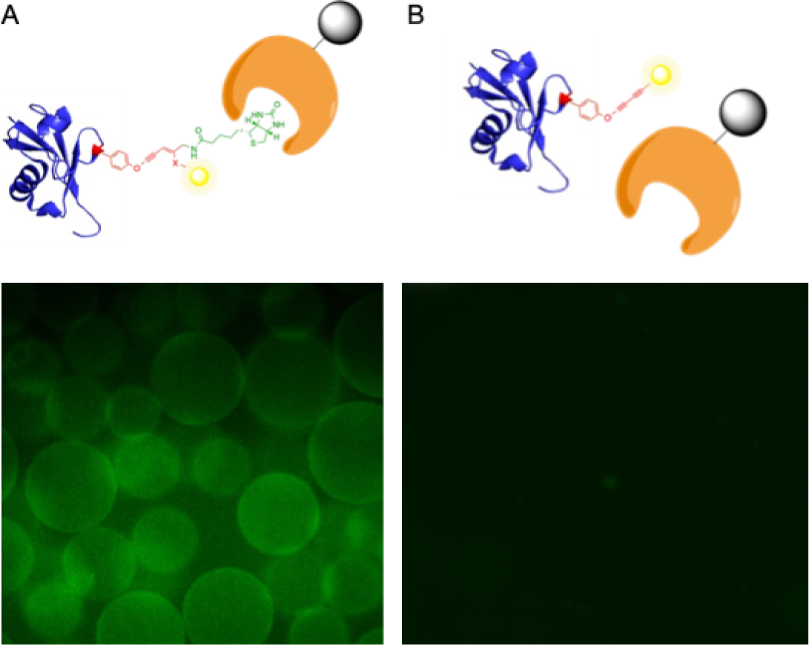
Streptavidin bead pull-down assay of protein bioconjugates. A) Incubation of the Ub-48-biotin-BODIPY conjugate with immobilized streptavidin resin. The fluorescent beads indicate the strong biotin association with the streptavidin resin and confirm the trivalent conjugation. B) Incubation of the Ub-48-AlexaFluor-488 conjugate with immobilized streptavidin resin. No fluorescence is observed due to the absence of biotin, demonstrating that the fluorescence observed in the trivalent conjugate is due to the presence of biotin and not nonspecific interactions of the conjugate with the beads.

While we developed this chemistry to afford multivalent conjugates via 1,3-diyne additions, we also became interested in broadening the utility of the approach by making divalent conjugates via thio-yne reactions with the initial *p*PrF terminal alkyne residue. While this is not a unique bioconjugation method and has been employed in the literature before, we were interested in ascertaining whether our optimized conditions were applicable to monoynes, as, to the best of our knowledge, no previous biological thio-yne reaction employed rongalite.^87–89^ Moreover, hydroselenation reactions have also not been previously reported, affording a more direct route to the incorporation of selenium into proteins. The GFP-151-*p*PrF (∼1 mg/mL, PBS pH 6) was combined with BODIPY FL l-cystine or synthesized FITC-diselenide (0.2 mM or 0.33 mM, respectively), rongalite (10 mM), and K_2_CO_3_ (6.6 mM) and reacted at room temperature in a manner analogous to the hydrothiolation of 1,3-diyne conjugates. After ∼24 h, the reaction was buffer-exchanged and analyzed by SDS-PAGE with fluorescence imaging. The hydrothiolation and hydroselenation of GFP-151-*p*PrF were found to proceed, albeit at a slower rate due to the decreased reactivity of a monoyne relative to a diyne (Figure 5). Interestingly, this reaction did not afford a divalent conjugate when reacted within a 6 h time frame of the diyne but required a full 24 h to yield product.

**Figure 5:**
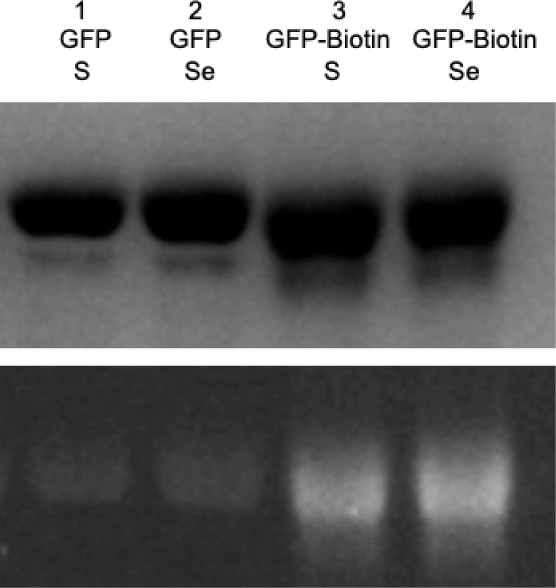
SDS-PAGE analysis of monoalkyne reactivity and diyne reactivity with the sulfur and selenium fluorophores after 24 h of reaction. Increased coupling is observed for diyne (GFP-biotin), as observed by increased fluorescence (bottom). Fluorescence is also observed with the terminal alkyne-containing protein (GFP) under identical conditions. No fluorescence is observed when reacting GFP with either fluorophore for 6 h. Coomassie staining (top) indicates no protein degradation and similar levels of protein loading.

## Conclusions

Overall, a new methodology has been developed for the biological hydrochalcogenation of polyynes to yield multivalent bioconjugates. Moreover, new conditions have been developed for the preparation of divalent conjugates through the addition of a chalcogen to a terminal alkyne using a rongalite-based system. This conjugation occurs at room temperature in a matter of hours without notable protein degradation. Enhanced via the incorporation of an ncAA, this conjugation is highly specific, involves minimal genetic perturbation, and can be employed to introduce novel functions into proteins. Ultimately, the increased valency of the conjugates can rapidly and efficiently afford enhanced therapeutic and diagnostic agents. Future work will aim to develop highly functionalized conjugates with more relevant proteins to fully illustrate the potential of this approach.

## Supplementary Material



## Notes

Additional experimental and synthetic details, control experiments, full SDS-PAGE gels of expression and conjugation results, and conjugate characterization (PDF)

## Abbreviations

ncAA: noncanonical amino acid
GFP: green fluorescent protein
Ub: ubiquitin
PBS: phosphate-buffered saline
FITC: fluorescein isothiocyante
*p*PrF: para-propargyloxyphenylalanine
