# Supplementary material for: Hydrochalcogenation Reactions with Noncanonical Amino Acids as a Route to Increase Bioconjugate Valency
Source: ACS Omega. 2025 Oct 2;10(40):47429–37. doi: 10.1021/acsomega.5c07015 (PMC12529139; doi:10.1021/acsomega.5c07015)
Supplement: Supplementary file 1 [file ao5c07015_si_001.pdf]

Supporting Information

Hydrochalcogenation reactions with non-canonical amino acids as a route to increase bioconjugate valency

Emily L. Boyt, Tyler L. Skeen, Cedrick R. Dimaranan, Evan M. London, Aaron S. Wang, Sophia K. Rothman, Milania G. Dehring, Alexander C. Willard, Emily M. Peairs, Elizabeth A. King, and Douglas D. Young*

Department of Chemistry, William & Mary, Williamsburg, VA 23185 USA

Experimental.

Synthesis of p-propargyloxyphenylalanine (pPrF).[1]

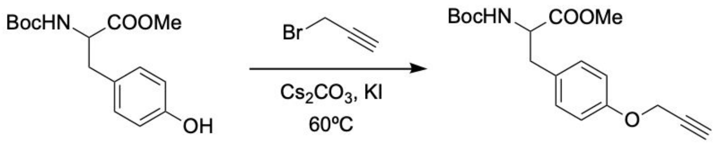

Boc-Tyrosine-OMe (0.500 g, 1.69 mmol) and cesium carbonate (0.826 g, 2.54 mmol) were added into a flame-dried vial with a magnetic stir bar and dissolved in dry DMF (10 mL). This mixture was then stirred at 60ºC for 30 minutes. Propargyl bromide (0.452 mL, 5.10 mmol) and a spatula tip of KI were then added to the vial. The reaction was allowed to stir at 60ºC overnight and then cooled to room temperature and filtered. Next, the filtered reaction mixture was diluted with brine (20 mL) and extracted with dichloromethane (3 x 20 mL). The organic layer was washed with brine (3 x 20 mL), dried over $MgSO_4$, and concentrated in vacuo. The resulting oil was purified via flash chromatography (25% EtOAc in hexanes) and concentrated to yield protected p-propargyloxyphenylalanine (pPrF) as a white crystal (0.464 g, 83% yield). ^{1}H NMR (400 MHz, $CDCl_3$): δ 7.02 (d, 2 H), 6.82 (d, 2 H), 4.95 (d, 1 H), 4.53 (d, 2 H), 4.27 (d, 1 H), 3.71

(s, 3 H), 3.02 (m, 2 H), 2.39 (t, 1 H), 1.41 (s, 9 H). ^{13}C NMR (400 MHz, $CDCl_3$): δ 172.4, 157.9, 130.3, 127.9, 114.5, 83.5, 79.9, 68.8, 66.0, 54.5, 52.2, 37.4, 28.3, 28.2, 21.1, 15.1

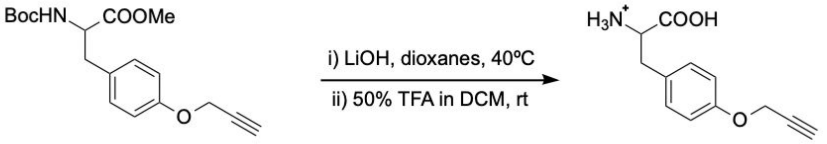

To remove the methyl protecting group, 3 mL of a 1:1 solution of 1M LiOH in water and dioxanes was added to protected pPrF (0.464 g., 0.73 mmol) in a vial on ice. The mixture was then stirred at room temperature for two hours and the dioxanes were removed in vacuo. The aqueous solution was cooled on ice and 6 M HCl was added dropwise until a pH of 4 was achieved and a solid white precipitate formed. The mixture was then extracted with cold ethyl acetate and the organic layer was washed with cold water. The organic layer was then dried over $MgSO_4$ and concentrated under reduced pressure to afford a yellow oil. This yellow oil was then dissolved in 50% trifluoroacetic acid in dichloromethane on ice and allowed to stir at room temperature for one hour. The solvent was removed in vacuo, affording the trifluoroacetate salt of pPrF as a brown solid (0.301 g., 98% yield). ^{1}H NMR (400 MHz, CD_3OD): δ 7.19 (d, 2 H), 6.93 (d, 2 H), 4.91 (s, 1 H), 4.14 (m, 1 H), 3.02 (m, 2 H), 2.54 (t, 1 H). ^{13}C NMR (400 MHz, $CDCl_3$): δ 168.4, 155.7, 128.6, 125.4, 113.5, 73.4, 54.4, 52.3, 45.4, 25.5.

Synthesis of FITC selenocystamine.[2]

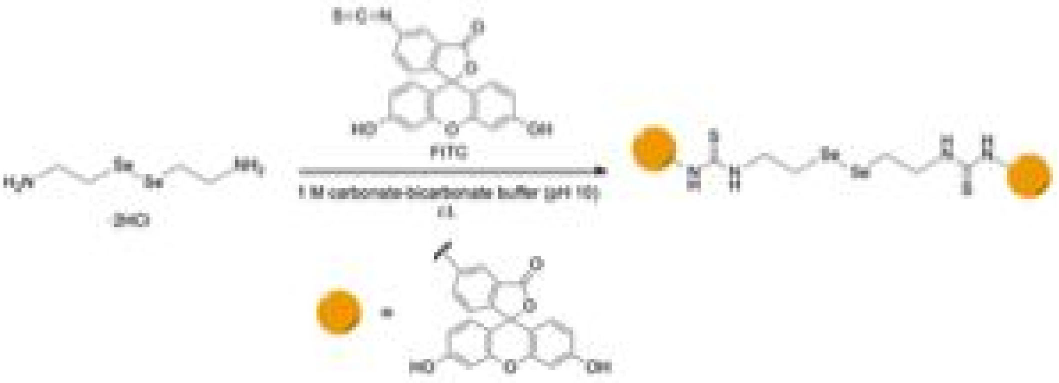

Fluorescein 5-isothiocyanate (FITC; 2.5 mg, 0.0065 mmol) was dissolved in DMF (130 µL) in an Eppendorf tube. In a separate Eppendorf tube, selenocystamine dihydrochloride (2.9 mg, 0.0090 mmol) was dissolved in carbonate-bicarbonate buffer (870 µL, 1 M, pH 10). The FITC solution (50 mM) was added dropwise to the selenocystamine dihydrochloride solution (10.4 mM). The reaction was allowed to shake at room

temperature for 72 hours. Reaction completion was confirmed via thin layer chromatography in 10% methanol and 5% glacial acetic acid in dichloromethane. Upon completion, the reaction was concentrated *in vacuo* and purified by silica gel pipette column chromatography (10% methanol and 5% glacial acetic acid in DCM) to furnish double FITC-labeled selenocystamine as a red-orange solid. Owing to the small scale on which the reaction was performed, the product could not be characterized by ^{1}H NMR and ^{13}C NMR. MS (ESI) $C_{46}H_{34}N_4O_{10}S_2Se_2$ Expected: 1024.86; Observed: 1024.92

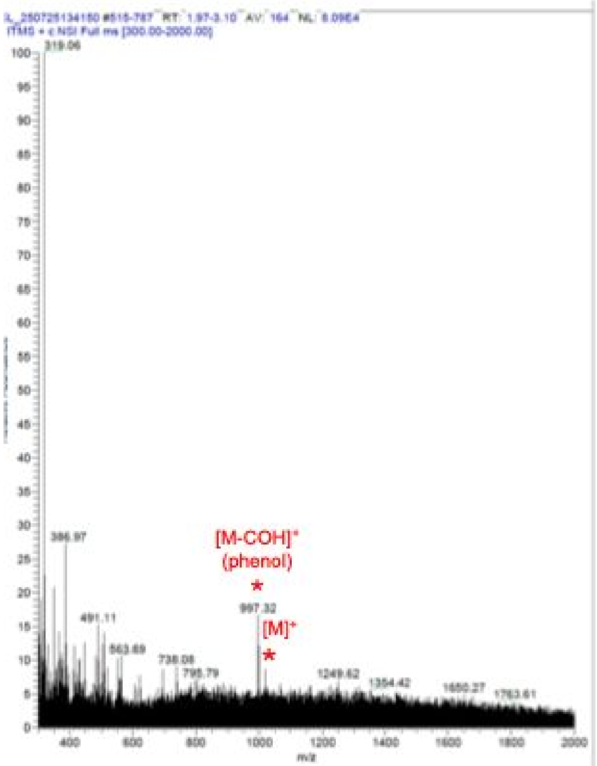

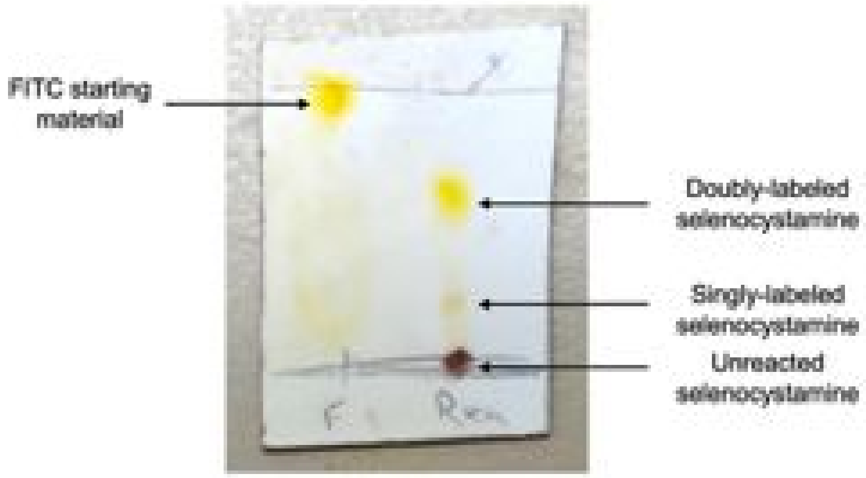

Figure S1. TLC analysis of the reaction of FITC and selenocystamine dihydrochloride. Ninhydrin staining of the TLC plate indicated that the reaction (right spots) produced both doubly-labeled selenocystamine (yellow, fluorescent spot that did not turn purple with ninhydrin) and singly-labeled selenocystamine (yellow, fluorescent spot that turned purplish with ninhydrin). Ninhydrin staining also revealed the presence of unreacted selenocystamine (purple spot that appeared with ninhydrin). No unreacted FITC was detected in the reaction mixture.

Figures.

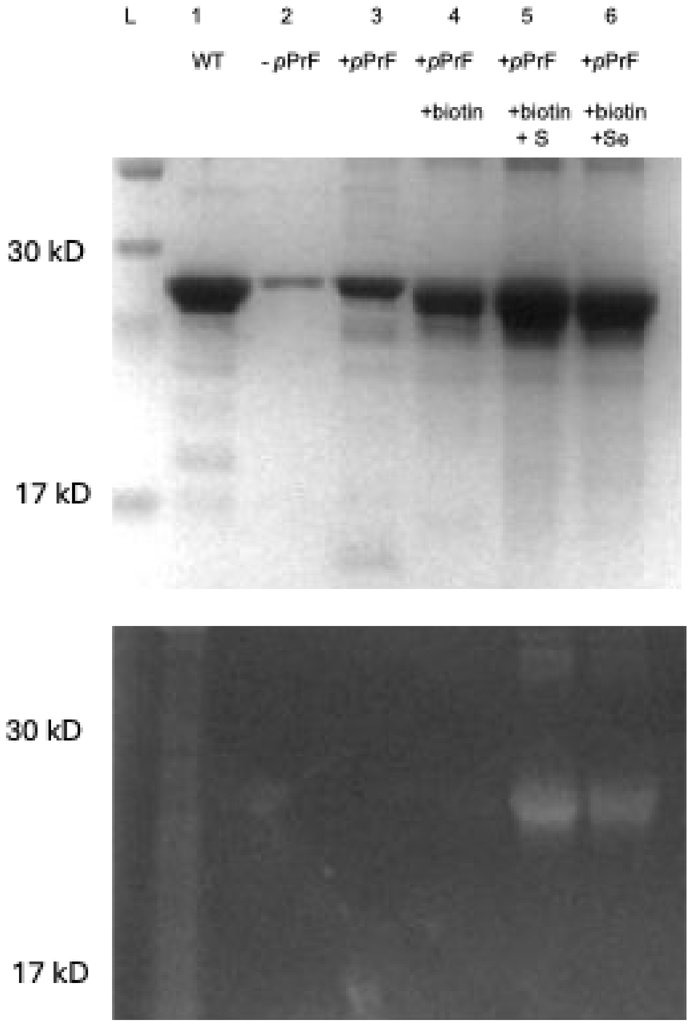

Figure S2. Denaturing SDS-PAGE of GFP expression and bioconjugation. Gel was imaged for both fluorescence (bottom) and Coomassie stained (top). Lane 1: Wild-type GFP; Lane 2: pEVOL-*p*CNF/GFP-Y151TAG expression in absence of ncAA (some tyrosine is incorporated in the absence of any ncAA in accordance to the literature);[3] Lane 3: pEVOL-*p*CNF/GFP-Y151TAG expression in presence of *p*PrF; Lane 4: Glaser-Hay bioconjugation of GFP-*p*PrF with biotin alkyne; Lane 5: Thio-yne addition of BODIPY-L-cystine to bivalent GFP-biotin conjugate; Lane 6: Hydroselenation of FITC-selenocystamine of bivalent GFP-biotin conjugate. Lanes 5 and 6 both demonstrate expected fluorescence following conjugation to the fluorophore, while other lanes lack fluorescence due to the expected denaturing of GFP.

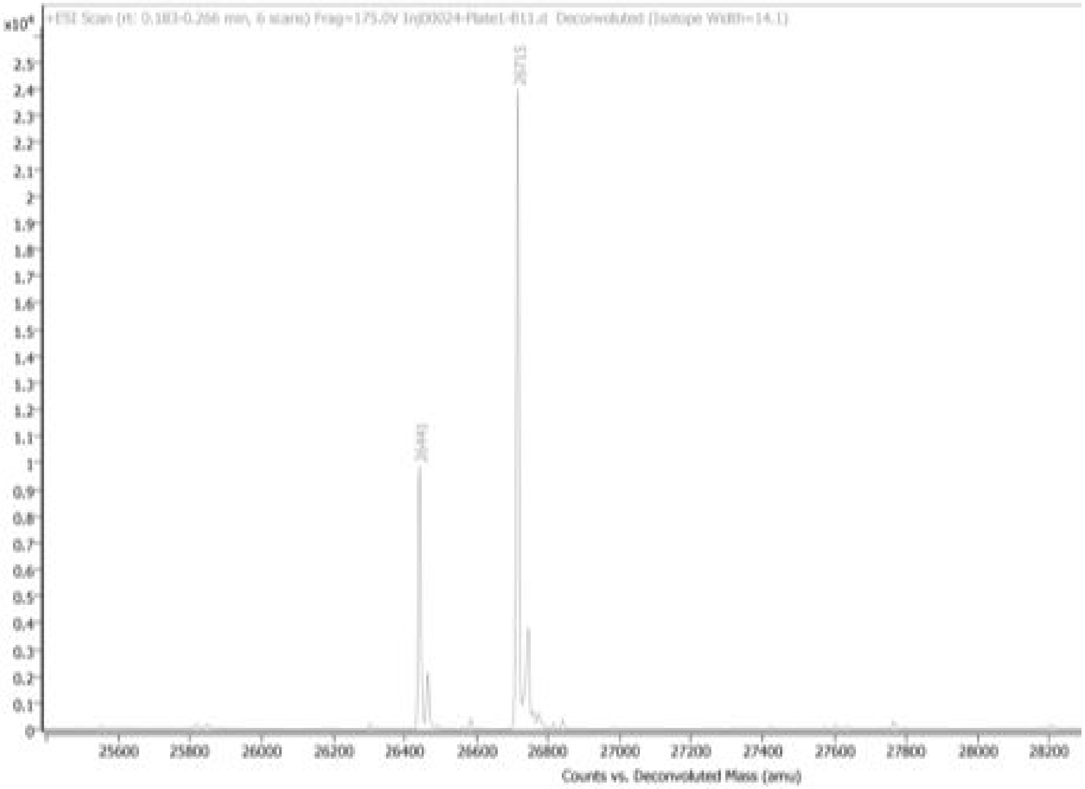

Figure S3. Mass spectrometry indicated the successful synthesis of a multivalent conjugate (observed m/z = 26715; expected m/z=26713) that contained a single biotin alkyne moiety and a single BODIPY-L-cystine moiety. The secondary peak is unreacted monoyne reacted with fluorophore to form the divalent conjugate GFP-fluorophore (m/z=26411). Samples were analyzed on an Agilent 6520 Accurate-Mass Quadrupole-Time-of-Flight (Q-TOF) mass spectrometer equipped with an electrospray (ESI) ionization source and liquid chromatography (LC) (Agilent). Ionization settings were: positive mode; capillary voltage 3500 kV; fragmentor voltage 200 V; drying gas temperature 350 °C. Instrument was set to standard 2 GHz, extended dynamic range and deconvolution was performed by Agilent MassHunter Qualitative Analysis software using the maximum entropy setting.

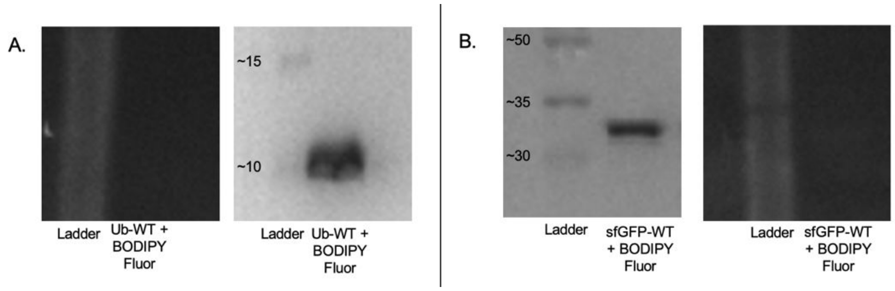

Figure S4 Incubation of wild-type ubiquitin (Ub) (**A.**) and wild-type sfGFP (**B.**) with rongalite, K_2CO_3, and BODIPY FL L-cystine did not result in a reaction**.**

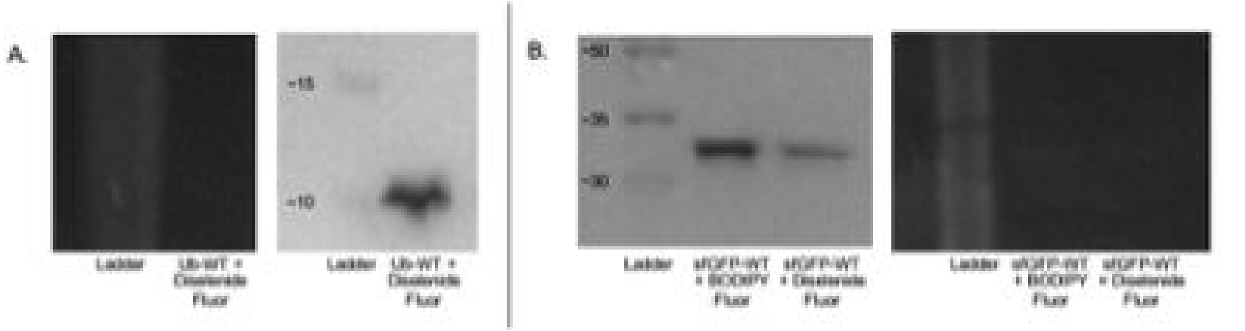

Figure S5. Incubation of wild-type ubiquitin (Ub) (**A.**) and wild-type sfGFP (**B.**) with rongalite, K_2CO_3, and diselenide fluorophore did not result in a reaction.

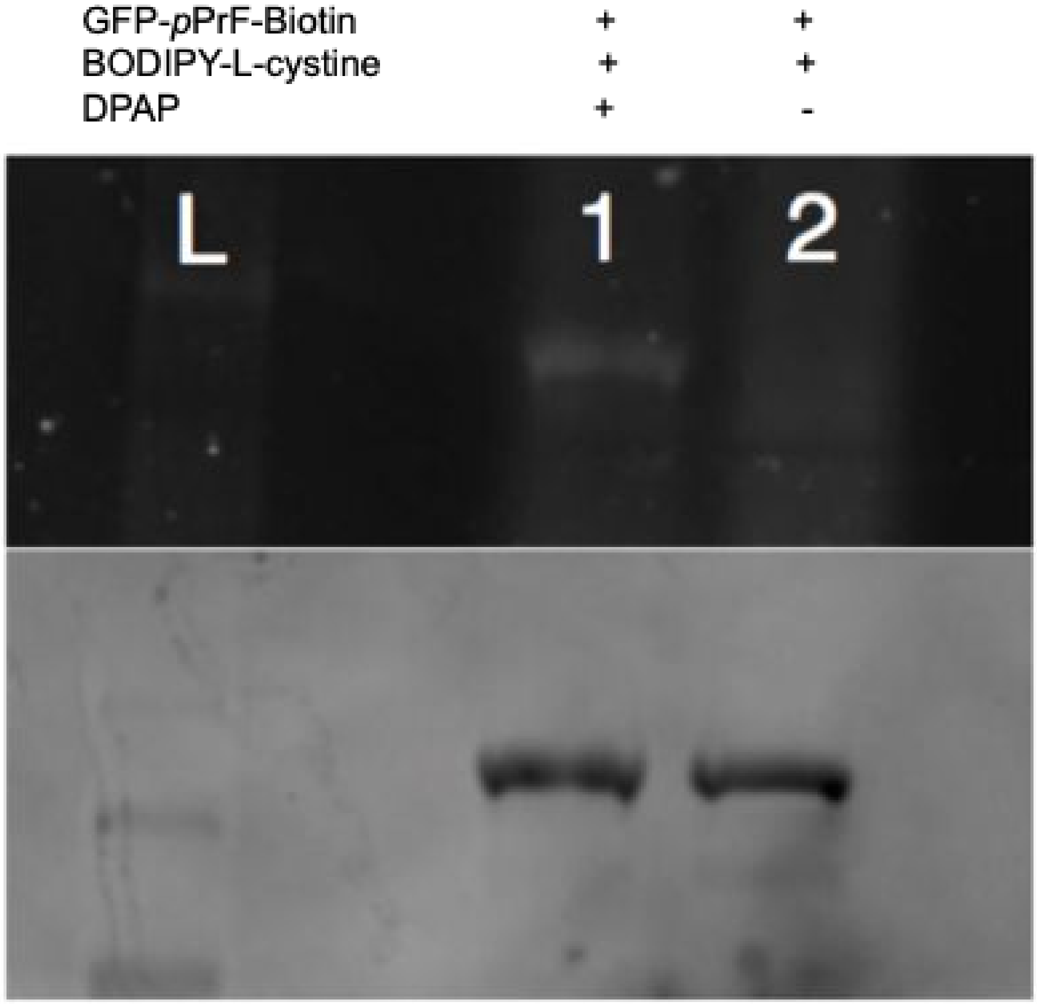

Figure S6. Initial experiments using photoactiviation catalysts DPAP (I2959 data not shown but identical). Incubation with the photoactivator and fluorophore resulted in low yields of conjugation (lane 1), whereas absence of DPAP did not result in thio-yne reaction (lane 2). Both samples were irradiated for 30 min at 365 nm. Coupling efficiencies could not be further optimized, resulting in the transition to rongalite conditions.

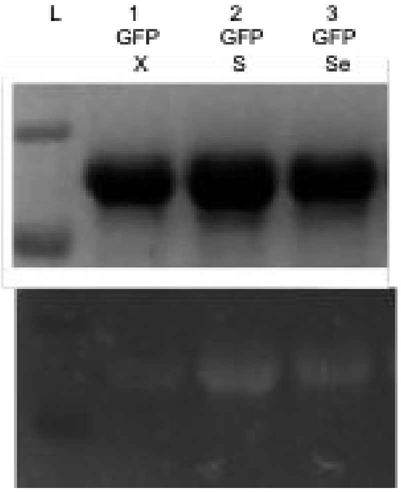

Figure S7. Monoyne rongalite reactions with fluorophores. A GFP-*p*PrF control (lane 1) was loaded with the same protein concentration as rongalite reactions (lanes 2 and 3) but was not subjected to reaction. The comparable Coomassie stained protein bands (top) in all three samples demonstrates that no protein degradation is observable by SDS-PAGE. The GFP-pPrF was reacted with either the thiol (lane 2) or selenide (lane 3) fluorophore and fluorescence was observed in these two lanes (bottom).

References

(1) Deiters, A.; Schultz, P. G. In vivo incorporation of an alkyne into proteins in Escherichia coli. *Bioorg Med Chem Lett* **2005**, *15* (5), 1521-1524. DOI: 10.1016/j.bmcl.2004.12.065.
(2) Landino, L. M.; Brown, C. M.; Edson, C. A.; Gilbert, L. J.; Grega-Larson, N.; Wirth, A. J.; Lane, K. C. Fluorescein-labeled glutathione to study protein S-glutathionylation. *Anal Biochem* **2010**, *402* (1), 102-104. DOI: 10.1016/j.ab.2010.02.006.
(3) Young, T. S.; Ahmad, I.; Yin, J. A.; Schultz, P. G. An enhanced system for unnatural amino acid mutagenesis in E. coli. *J Mol Biol* **2010**, *395* (2), 361-374. DOI: 10.1016/j.jmb.2009.10.030.
